# Development of a Plastic-Based Microfluidic Immunosensor Chip for Detection of H1N1 Influenza

**DOI:** 10.3390/s120810810

**Published:** 2012-08-06

**Authors:** Kyoung G. Lee, Tae Jae Lee, Soon Woo Jeong, Ho Woon Choi, Nam Su Heo, Jung Youn Park, Tae Jung Park, Seok Jae Lee

**Affiliations:** 1 Center for Nanobio Integration & Convergence Engineering (NICE), National NanoFab Center, 291 Daehak-ro, Yuseong-gu, Daejeon 305-806, Korea; E-Mails: kglee@nnfc.re.kr (K.G.L.); tjlee@nnfc.re.kr (T.J.L.); swjeong@nnfc.re.kr (S.W.J.); hwchoi@nnfc.re.kr (H.W.C.); 2 Bioprocess Engineering Research Center, KAIST, 291 Daehak-ro, Yuseong-gu, Daejeon 305-701, Korea; E-Mail: hns0924@kaist.ac.kr; 3 Biotechnology Research Division, National Fisheries Research & Development Institute (NFRDI), 408-1 Sirang-ri, Gijang, Busan 619-705, Korea; E-Mail: jypark@nfrdi.go.kr; 4 Department of Chemistry, Chung-Ang University, 84 Heukseok-ro, Dongjak-gu, Seoul 156-756, Korea

**Keywords:** immunosensor, H1N1 influenza, microfluidics, plastic chip

## Abstract

Lab-on-a-chip can provide convenient and accurate diagnosis tools. In this paper, a plastic-based microfluidic immunosensor chip for the diagnosis of swine flu (H1N1) was developed by immobilizing hemagglutinin antigen on a gold surface using a genetically engineered polypeptide. A fluorescent dye-labeled antibody (Ab) was used for quantifying the concentration of Ab in the immunosensor chip using a fluorescent technique. For increasing the detection efficiency and reducing the errors, three chambers and three microchannels were designed in one microfluidic chip. This protocol could be applied to the diagnosis of other infectious diseases in a microfluidic device.

## 1. Introduction

Swine-origin influenza virus, a high-risk human influenza A virus (H1N1), is a serious health threat and potential leading cause of death all around the World [1]. The World Health Organization (WHO) has reported that more than 16,000 cumulative deaths were reported from 213 countries due to H1N1 in February 2010 [2]. Several laboratory diagnostic methods have been developed to monitor the outbreaks of the virus as follows: (1) specific real-time polymerase chain reaction (PCR)-based detection method, (2) isolation of H1N1 influenza virus, (3) detection of 4-fold rise of neutralization antibodies to the virus [3,4]. However, these methods require highly skilled-personnel and expensive laboratory instruments. In addition, they are not suitable for undeveloped countries because of the limited access to central laboratories and expensive costs.

To overcome these issues, microfluidic immunoassay systems have been introduced because of their various advantages, including high throughput, high-efficiency, low-cost and minimized consumption of samples and reagents [5]. After the development of soft lithography techniques using poly(dimethylsiloxane) (PDMS), PDMS has become the most popular microfluidic device materials and offers several advantages such as easy handling, good sealing properties and high optical transparency [6]. However, the poor chemical stability in different types of organic solvents, difficulty in surface modification and mass production have limited the use of PDMS in the various applications [7].

Recently, because of the material issues, some researchers have been attempted to use plastic materials as an alternative solution. Among the various types of polymers, cyclic olefin copolymer (COC) is one of the most popular polymeric materials in the fabrication of microfluidic chips. COC is a well-known polymeric material with various advantages, including high clarity and light transmission, excellent mechanical properties and great biocompatibility [8].

Furthermore, effective immobilization of proteins is essential and important in microfluidic chips to be used as immunosensors. Several methods to immobilize antibodies on the sensor chip surface have been developed, including physical adsorption, covalent binding, and specific interaction between avidin and biotin [9,10]. However, these previous methods have limitations in terms of denaturization, extra chemical modification and random orientation. In order to overcome these issues, Brown *et al.* and Park *et al.* developed specific gold-binding polypeptide (GBP) that endows the orientation of proteins in their functional state [11,12]. GBP shows a strong binding affinity to the gold surface without any surface modifications [13–15]. Therefore, GBP-fusion proteins could be selectively and functionally immobilized onto the gold surface.

In this study, we carefully designed microfluidic devices, and the surface of a detection chamber was coated with gold for the direct assembly of proteins. A microfluidic-based immunosensor to detect human H1N1 influenza was developed into a low-cost immunosensor based on the exploration of fluorescence signals. The detection of a specific antibody among serological assays in blood samples was performed in the microfluidic biosensor chip by immunoreactions between the GBP-recombinant influenza hemagglutinin antigen (GBP-H1a) fusion protein and its specific antibody (Ab). The GBP-H1a fusion protein as a bioreceptor and the fluorescence-labeled Ab as a marker were used to provide an excellent detection signal. In addition, the chip fabrication and sensing characteristics are reported in detail.

## 2. Experimental Section

### 2.1. Reagents and Materials

COC was purchased from TOPAS Advanced Polymers (Frankfurt-Höchst, Germany). Unless otherwise stated, all chemicals were purchased from Sigma-Aldrich (St. Louis, MO, USA) and used without further purification steps. Rabbit anti-H1 polyclonal Ab was obtained from AbFrontier (Seoul, Korea).

### 2.2. Design and Fabrication of Microfluidic Device

The microfluidic immunosensor device is fabricated by the injection molding method. The master stamp was fabricated using a micromilling process. The size and thickness of the stamp were 95 × 95 mm and 1.2 mm, respectively. We used a plastic microinjection mold machine (A270C 400-100, ARBURG, Lossburg, Germany) to produce the microfluidic device in order to achieve cost-effectiveness and facilitate mass production. Once the chip was fabricated, chromium (50 Å) and gold (100 Å) were coated on the detection area for further immobilization of GBP-H1a. In this work, the ultrasonic bonding method with an ultrasonic bonder (2000X, Branson, Danbury, CT, USA) was employed to bond the COC chips. For this purpose a melting line with a height of 20 μm was made around microchannels. Once the ultrasonic energy was applied to the COC plates, the sonic energy was intensively localized on the top of the melting line. After the lines were immediately melted and then cooled, both COC plates were tightly bonded with each other to fabricate the plastic-based microfluidic device.

### 2.3. Preparation of GBP-H1a Fusion Protein

Bifunctional fusion protein was created by genetically fusing GBP and H1a, allowing specific interactions between GBP and the gold substrates as well as the capture of H1a and its antibodies. As described in the previous report [16], the DNA fragments encoding the H1N1 viral surface antigen (H1a) were amplified by PCR with forward primer (5′-CCATGGCATATGGG CCACCATCACCATCACCACGGCAA-3′) and reverse primer (5′-CCGCTCGAGCTGGCTACG CACTTTTTCATACAGGTTTTTAACGTTGCTATCGTGATAGCCGCAAGCTTGTCGACA-3′) for the construction of 6His-GBP-H1a fusion gene. Then, the PCR product was cloned into the *Nde*I-*Xho*I fragment of pET-6HGBP to make pET-6HGBP-H1a.

Recombinant *E. coli* BL21 (DE3) strain harboring pET-6HGBP-H1a was cultivated in Luria-Bertani (LB) medium (10 g/L bacto-tryptone, 5 g/L yeast extract, and 5 g/L NaCl) supplemented with 100 μg/mL of ampicillin at 37 °C and 250 rpm. At OD_600_ (DU600^®^ Spectrophotometer, Beckman Coulter, Brea, CA, USA) of 0.4, cells were induced with 1 mM of isopropyl-*β*-D-thiogalactopyranoside (IPTG, Sigma) for the production of the fusion protein. After induction, cells were further cultured for 4 h. The cells were then harvested and disrupted by sonication (Braun Ultrasonics, Orlando, FL, USA) for 1 min at 20% output power. After centrifugation at 16,000× g for 10 min at 4 °C, the pellet containing the soluble protein fraction with high-level expressed target fusion-protein was collected for purification of the fusion protein. Because of the 6His tags, a HisTrap™ column (GE Healthcare, Chalfont St. Giles, UK) was used to purify the fusion protein without further purification steps. The protein concentration was determined by the Bradford assay with bovine serum albumin (BSA) as a standard.

### 2.4. Surface Plasmon Resonance (SPR) Analysis

The binding of the GBP-H1a fusion protein onto the surface of a SPR bare gold chip was characterized by SPR measurement using a BIAcore3000™ with an automatic flow injection system (Biacore AB, Uppsala, Sweden). All experiments were performed in phosphate-buffered saline (PBS, pH 7.4) at room temperature. A fresh SPR sensor chip was attached to a separate chip carrier for easy assembly in the SPR system. After docking and priming of the SPR chip, PBS was used to flush the activated surface and thereby minimize non-specific binding and any unbound sites by removing loosely bound material and dust. All samples were injected onto the gold chip surface at a flow rate of 5 μL/min for 10 min at room temperature using a liquid-handling micropipette in the SPR system. The surface was then washed and equilibrated using PBS. The GBP-H1a fusion protein (50 μL of a 25 μg/mL solution) was injected onto the SPR chip surface. Before binding anti-H1 Ab, 50 μL BSA of 100 μg/mL was injected to block non-specific binding for 10 min. All SPR sensorgrams were fitted globally using BIA evaluation software (Biacore).

## 3. Results and Discussion

**Figure 1.**
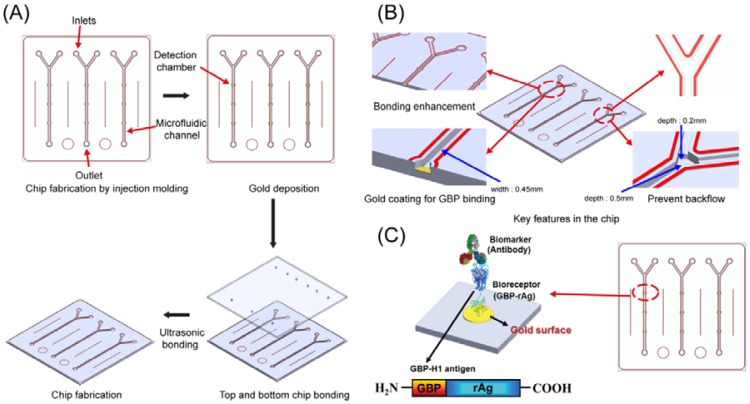
Schematic illustration for (**A**) the fabrication of COC-based microfluidic chip, (**B**) detailed features of the chip, (**C**) GBP-H1a fusion protein immobilization step and Cy3-labeled Ab reaction in the chip.

The plastic-based microfluidic devices were fabricated by an injection molding technique. The microchannels-embedded master mold was obtained by a milling technique, and it was used for casing the microfluidic devices. In this case, COC was chosen as a substrate material to produce the device due to its great advantages over the other thermoplastic polymers in terms of optical, physical and chemical properties and biocompatibility. The total cycle time of the microinjection molding process took less than 1 min to replicate the COC substrate. After location of the master mold in the injection mold machine, COC was injected through the nozzle, and the microfluidic device was released from the mold. The production process and conditions are similar to the previous research which demonstrated various microinjection conditions to form microstructures [17]. Holes of 1 mm in diameter for an inlet and outlet ports were punched to load the sample and buffer solutions into the microchannels. As shown in Figure 1, the channel depths are 200 and 500 μm with 450 μm in width, respectively. The diameter of the detection chamber is 1 mm, and it was coated with chromium and gold by sputtering for the immobilization of GBP-H1a fusion protein. The design and fabrication process of microfluidic device are described in Figure 1(A). The key features of microstructures including welding lines, gold deposition on detection chamber, and backflow prevention structures are schematically demonstrated in Figure 1B. Previously, thermal bonding method has been popular to bond plastic-based microfluidic device. However, this method requires high temperature and takes too much time to bond it. Recently, an ultrasonic bonding method has been developed as an alternative solution to the thermal bonding. However, welding line is essential for bonding between plastic chips in this technique. In this experiment, we carefully designed the welding lines as shown in red color in Figure 1(B), and these lines were melted during the ultrasonic bonding process for the sealing of the chips. After coating of gold, two COC plates were placed on the ultrasonic bonder (2,000X, Branson) and set the bonder in time mode with weld frequency of 20 kHz. After the setting of the mode, the COC was bonded as following conditions: (1) 800 Pa weld pressure, (2) 0.2 s of weld time, (3) 75% of amplitude, (4) 10 s of hold time, (5) 1.5 kPa of hold pressure. Occasionally, backflow of the buffer or target solutions occurs in microfluidic channels during the injection to cause a contamination problem. Therefore, there would be required a unique microstructure in a microchannel to prevent backflow of solution and contamination of microchannel. Among various methods, two different channel depths were applied into this device as shown in Figure 1(B). This method was worked properly due to the pressure difference in the microchannel to prevent the backflow.

The integrated microfluidic immunosensor chips can reduce the analytical time compared to the conventional methods. In addition, usage of plastic facilitates low-cost mass production of disposable and easy-to-use microfluidic chips. Previously reported plastic-based immunosensors required a surface modification step for the immobilization of biomolecules. Among these methods, silanization using 3-aminopropyltriethyoxysilane (APTES) is a widely used method for coupling of biomolecules onto the inorganic substrate [18]. However, silane is difficult to react with organic materials without any hydroxyl groups present on the surface by using UV/ozone treatment [5]. In order to overcome these issues, a specially engineered peptide was developed and used to immobilize onto the specific targeted surface, especially gold. There is no requirement of complicated steps for coupling of antibody or antigen. The GBP-H1a fusion protein can be simply and selectively immobilized in the microchannel on the gold surface as described in Figure 1(C).

**Figure 2.**
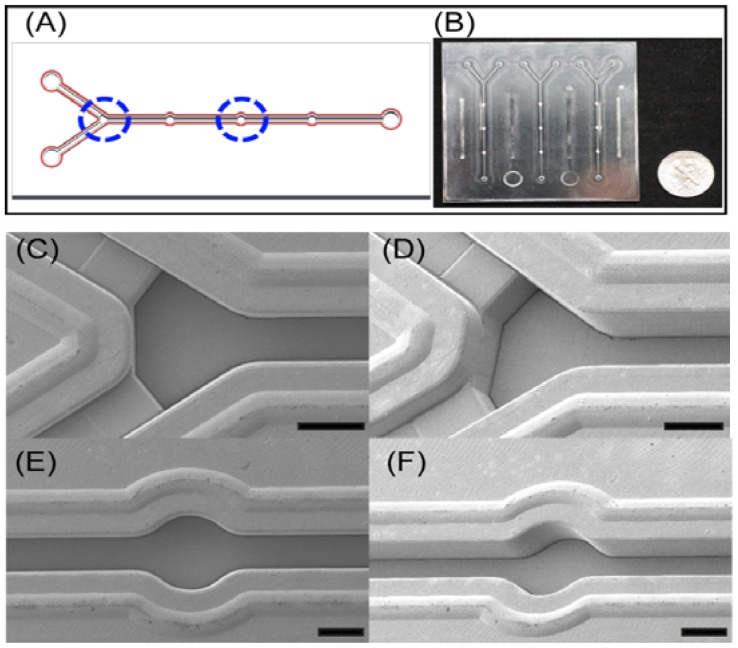
(**A**) Schematic illustration of microfluidic device and (**B**) its picture. SEM images of the (**C**–**D**) Y-junction and (**E**–**F**) detection chamber. All SEM images were shown in both top angle and tilted angle, and inserted scale bars are 500 μm.

In this device, Y shaped inlet channels with backflow-prevented microstructure and detection chambers were designed for an efficient immunoassay as shown in Figure 2. The key features of microstructures in microfluidic device were successfully replicated using the microinjection molding, and they were confirmed through top and tilted scanning electron microscopy (SEM, Hitachi S4800, Ibaraki, Japan) images as shown in Figure 2(C–F). Due to the height differences between inlet channels and main channels (Figure 2(C,D)), this could prevent the backflow of the solutions. This device is composed of three detection chambers with 1 mm in diameter for the further immobilization of GBP-H1a fusion protein as shown in Figure 2, and these chambers may reduce the errors during analysis by averaging signals. All protuberant microstructures near to the microchannels were specially designed as welding lines. During the ultrasonic bonding process to bond the top and bottom of the microfluidic chip, these lines would be melted by concentrating the ultrasonic energies on the top of welding lines.

**Figure 3.**
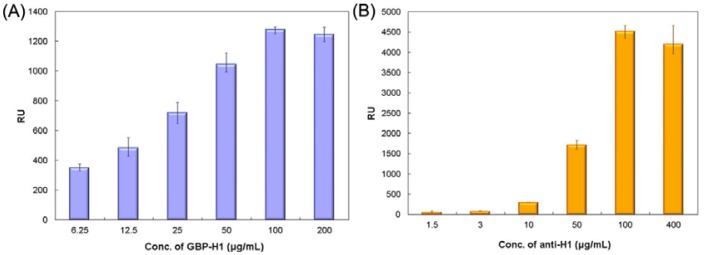
Molecular binding optimization of GBP-H1a fusion protein and its specific Ab with different concentrations. (**A**) Specific bindings of GBP-H1a fusion protein onto the gold surface. (**B**) Subsequent bindings of anti-H1 Ab. The bindings were started at 150 s and the unbound samples were washed at 750 s.

To investigate the sensing window of the fusion protein, different concentrations (6.25, 12.5, 25, 50, 100 and 200 μg/mL, respectively) of GBP-H1a fusion protein were immobilized onto the gold chip surface by surface plasmon resonance (SPR) microfluidics. A greater shift in resonance unit (RU) was observed by SPR analysis with increasing concentration of immobilized GBP-H1a fusion protein bound on the planar surface at various concentrations as shown in Figure 3(A). These results suggest that the SPR sensor with GBP-fusion protein implemented on the gold surface can be an effective system for biomolecular immobilization. Furthermore, the concentration of GBP-H1a fusion protein was fixed to 100 μg/mL due to its best immobilization concentration.

For the subsequent binding of anti-H1 Ab, different concentrations (1.5 to 400 μg/mL) of specific antibody were bound to the GBP-H1a fusion protein on the gold sensor chip. The saturated 4,000 RU value obtained with SRP experiments implies that about 4 ng of anti-H1 Ab was immobilized onto the gold surface area of 1 mm^2^. One RU is determined as 0.0001° of resonance angle shift and equivalent to a mass change of the 1 pg/mm^2^ on the SPR sensor chip surface [19,20]. Specific anti-H1 Ab against the H1 influenza surface antigen, which is a hemagglutinin subunit having a high immunogenicity and surface probability, was applied to the GBP-H1a fusion protein-layered surface to monitor specific binding between GBP-H1a fusion protein and anti-H1 Ab by SPR biosensor as shown in Figure 3(B).

**Figure 4.**
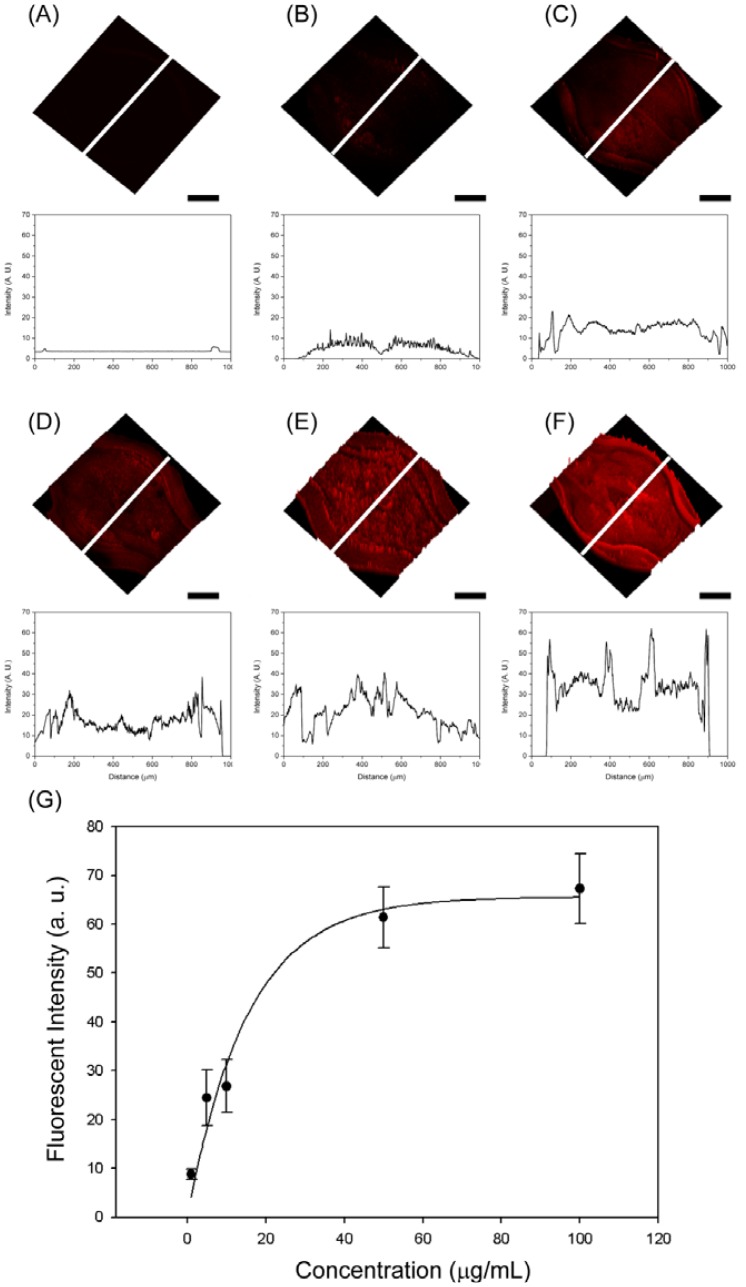
Fluorescent confocal microscopy images and their corresponded line profiles scanned through the center line (white color) of (**A**) control, (**B**) 1, (**C**) 5, (**D**) 10, (**E**) 50, and (**F**) 100 μg/mL Cy3-labeled Ab immobilized on the gold surface, respectively. Scale bars represent 200 μm. (**G**) Calibration plot for the sandwich immunoassay in a plastic-based microfluidic immunosensor chip.

To further investigate whether this microfluidic device can be used in immunoassay, we employed GBP-H1a fusion protein, BSA as a blocking agent and Cy3-labeled anti-H1 Ab. Cy3-labeled anti-H1 Ab is a strongly fluorescent molecule, and the fluorescence-based immunoassay is more sensitive compared to the most colorimetric assays in most of the cases [5]. The whole immunosensing process was carried out by using COC microfluidic chips at room temperature. The three detection chambers are included in one microchannel to verify the sensing results which may reduce the error of the signal. In addition, one chip is composed of three different detection zones to test different concentrations of the target Abs. First of all, 100 μg/mL of GBP-H1a fusion protein was injected through the microchannel for the immobilization on the surface of gold surface. After immobilization for 1 h, all the chips were washed with PBS solution and BSA solution (1 mg/mL) was injected to the channels to prevent the non-specific binding then washed with PBS solution. After the blocking and washing process, five different concentrations of Cy3-labeled-Ab (100, 50, 10, 5, and 1 μg/mL, respectively) were injected through the microchannel and left them for 1 h. After incubation for the further reaction, all immunosensors were rinsed with PBS solution three times, and the microchannels were blown off by air. All microfluidic immunosensor chips were examined under same conditions of confocal microscopy (Carl Zeiss LSM510 Meta NLO, Göttingen, Germany) as shown in Figure 4. At the low concentration of Cy3-labeled Ab applied, small fluorescent signals were observed in the detection chambers. As increasing the concentration of Ab, the whole detection chamber was covered with red fluorescence, and the signal intensities were also increased. In order to compare the signal intensity, the intensity profiles were also recorded because the fluorescence intensity is directly proportional to the amount of Cy3-labeled Ab attached to the surface of detection chamber. The fluorescence intensity changes at the center of chamber were measured, and their fluorescent images with same scale in Y-axis were showed. The fluorescent intensity graphs which correspond to each inserted white line also showed similar signal changing patterns compared with the fluorescent image. From these results, the specific binding of GBP-H1a was successfully immobilized on the gold surface, and the fluorescent images and emission profiles were subsequently increased due to the effective binding of Cy3-labeled anti-H1 Ab, which could be applicable in immunoassay onto the microfluidic chip surface.

As a result, the fluorescent intensity depends on the number of immobilized Cy3-labeled-Ab in the detection chamber under the same incubation period. In addition, all detection chambers were analyzed using the line profiles. As increasing the amount of Ab, the intensity of line profiles also increases over the time, and the intensity line is getting increased, which is properly matching with fluorescence images. From the analyzed fluorescence results, the calibration curve, which represents the relationship between the fluorescence intensity and Cy3-labeled-Ab, is shown in Figure 4(G). The regression equation could be expressed as:

(1) $$I=65.16\times (1-0.937^{C})$$

where I is the fluorescence intensity and C is the concentration (μg/mL) of Cy3-labeled-Ab with a correlation coefficient of 0.983.

## 4. Conclusions

In this study, we report a development of a microfluidic device for the detection of human influenza by antigen-antibody interaction based on a highly transparent and inexpensive polymer. The significant fluorescence intensity changes over the different concentrations to the serological antibodies and three different chambers in one microchannel provide more accurate information to detect the H1N1 flu virus. In addition, the immunosensor chips were successfully applied for the detection of the H1N1 without any surface modification of microfluidic chip. The proposed integrated plastic-based microfluidic chip could provide a significant improvement in the miniaturization and a cost-effective way for bio-analysis systems. Therefore, this platform offers perspective for point-of-care testing diagnosis in various infectious disease areas.
